# Activation of AMPKα mediates additive effects of solamargine and metformin on suppressing MUC1 expression in castration-resistant prostate cancer cells

**DOI:** 10.1038/srep36721

**Published:** 2016-11-10

**Authors:** SongTao Xiang, QiuHong Zhang, Qing Tang, Fang Zheng, JingJing Wu, LiJun Yang, Swei Sunny Hann

**Affiliations:** 1 Department of Urology Surgery, Guangdong Provincial Hospital of Chinese Medicine, The Second Clinical Medical Collage, Guangzhou University of Chinese Medicine, Guangzhou, Guangdong Province, 510120, China; 2 Department of Medical Oncology, Guangzhou, University of Chinese Medicine, Guangzhou, Guangdong Province, 510120, China

## Abstract

Prostate cancer is the second most common cause of cancer-related deaths worldwide. The mucin 1 (MUC1) oncoprotein is highly expressed in human prostate cancers with aggressive features. However, the role for MUC1 in occurrence and progression of castration-resistant prostate cancer (CRPC) remained elusive. In this study, we showed that solamargine, a major steroidal alkaloid glycoside, inhibited the growth of CRPC cells, which was enhanced in the presence of metformin. Furthermore, we found that solamargine increased phosphorylation of AMPKα, whereas reducing the protein expression and promoter activity of MUC1. A greater effect was observed in the presence of metformin. In addition, solamargine reduced NF-κB subunit p65 protein expression. Exogenously expressed p65 resisted solamargine-reduced MUC1 protein and promoter activity. Interestingly, exogenously expressed MUC1 attenuated solamargine-stimulated phosphorylation of AMPKα and, more importantly reversed solamargine-inhibited cell growth. Finally, solamargine increased phosphorylation of AMPKα, while inhibiting MUC1, p65 and tumor growth were observed *in vivo*. Overall, our results show that solamargine inhibits the growth of CRPC cells through AMPKα-mediated inhibition of p65, followed by reduction of MUC1 expression *in vitro* and *in vivo*. More importantly, metformin facilitates the antitumor effect of solamargine on CRPC cells.

Prostate cancer is the most common malignancy and the second most common cause of cancer-related death in men worldwide^1^. The survival rates remain poor especially for castration-resistant prostate cancer (CRPC) patients in spite of multiple treatment strategies, including surgery, chemo-radiation, endocrine, and targeted therapies^2^,^3^. Although the etiology of prostate cancer is still unclear, a large body of evidence has indicated that environmental factors and dysfunction in multiple genes are associated with the development and progression of this malignancy^4^,^5^. Despite of the advancement in understanding the molecular biology and therapeutic approaches, CRPC still harbors a significant amount of treatment difficulty with less effective therapeutic strategies available^6^,^7^. Therefore, search for the novel therapeutic approaches based on various combinations of medications to enhance the therapeutic efficacy are urgently desired.

Natural phytochemicals derived from dietary sources and medicinal plants have gained significant recognition in the control of carcinogenesis, and been considered as an ideal approach in prevention and treatment of cancer. Solamargine, one of major compounds of Solanum lycocarpum fruit glycoalkaloid extract and a major steroidal alkaloid glycoside purified from Solanum nigrum L (SNL), a traditional Chinese medicinal herb, has been shown to have anti-tumor activity against the several types of cancers^8^,^9^,^10^,^11^. Solamargine (SM) augmented trastuzumab and epirubicin-induced deaths of human lung cancer cells^12^. Also, solamargine reported to inhibit migration and invasion of hepatocellular carcinoma cells through reduction of matrix metalloproteinase (MMP) expression^13^. However, limited data have so far unveiled the links of this agent to prostate cancer treatment. The mechanisms and potential nontoxic benefits by which this agent affects prostate cancer survival remain unknown.

AMP-activated protein kinase (AMPK) is crucial cellular energy sensor and a key regulator of multiple metabolic pathways, which induces the catabolic processes that produces ATP and inhibits the anabolic ATP-consuming processes associated with a number of beneficial effects, such as decrease of inflammatory responses and retard of disease progression including diabetes and obesity, among others^14^,^15^. AMPK signaling also involves in growth, differentiation and progression of cancer, and has emerged as an attractive therapetic target to various types of cancer^16^,^17^,^18^. However, conflicting reports about its cellular functions in cancer have been reported. AMPK has been shown to induce progression and promote cancer cell survival in the face of extrinsic, intrinsic stimuli, and metabolic stress such as hypoxia and glucose deprivation^19^,^20^,^21^,^22^. Thus, the two distinct roles of AMPK acting as a tumor suppressor or an oncogene was depending upon the different environmental contexts, and whether AMPK should be targeted for activation or inhibition during cancer therapy, require clarification. Nevertheless, we need to consider the effects of tissue/tumor types, and the effectors of short/long-term AMPK signaling when investigating its role in associating with cancer.

Cell surface-associated mucin 1 (MUC1), a glycosylated transmembrane protein, is highly expressed in various malignant tumors and is associated with cellular growth, invasion, metastasis^23^,^24^. MUC1 was also highly expressed in the CRPC cells, and a potential correlation between functional androgen receptor (AR) signaling and MUC1 expression was observed^25^,^26^. MUC1 has been shown to bind to transcriptional factor, such as nuclear factor NF-kappaB (NF-κB)/p65, and promote downstream target gene expression^23^. We recently showed that curcumin inhibited growth of CRPC cells through multiple kinase-mediated inhibition of MUC1 protein^27^. However, the expression and functional significance of MUC1 gene in occurrence and progression of CRPC still remains poorly understood.

In this study, we explored the potential mechanism by which solamargine alone and combination of solamargine and metformin in the inhibition of CRPC cells. We provided the evidence demonstrating that solamargine inhibited the growth of CRPC cells through AMPKα-mediated inhibition of p65, followed by reduction of MUC1 expression *in vitro* and *in vivo*. More importantly, metformin enhanced the antitumor effects of solamargine on CRPC cells.

## Results

### The effects of solamargine and metformin on the growth of CRPC cells

**Figure 1:**
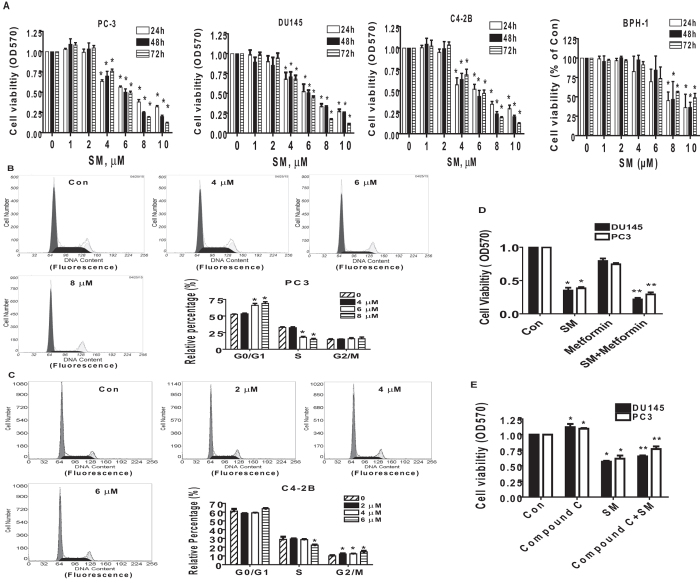
The effect of solamargine and metformin on growth of CRPC cells. (**A**) CRPC cell lines (PC3, DU145 and C4-2B) and one human benign prostate hyperplasia epithelial cell line (BPH-1) were treated with increased concentrations of solamargine for up to 72. Afterwards, the cell viability was determined using the MTT assay as described in the Materials and Methods Section. (**B**,**C)**, Prostate cancer cells PC3 and C4-2B were treated with increased concentrations of solamargine for up to 48 h. Afterwards, the cells were collected and processed for analysis of cell cycle distribution by flow cytometry after propidium iodide (PI) staining. And the percentages of the cell population in each phase (G0/G1, S and G2/M) of cell cycle were assessed by Multicycle AV DNA Analysis Software. Data are expressed as a percentage of total cells. Values are given as the mean ± SD from 3 independent experiments performed in triplicate. (**D**) PC3 and DU145 cells were treated with solamargine (6 μM) and metformin (5 mM) for 48 h. Afterwards, the cell viability was determined using the MTT assay. (**E**) PC3 cells were treated with compound C (10 μM) for 2 h before exposure the cells to solamargine (6 μM) for up to 48 h. Afterwards, the cell viability was determined using the MTT assay. *indicates significant difference as compared to the untreated control group (P < 0.05). **Indicates significant difference from solamargine treated alone (P < 0.05).

We stated to examine the effect of solamargine on growth of CRPC cells. We showed that solamargine inhibited the growth of two CRPC cell lines (PC3 and DU145) in the dose-dependent manner with significant effect observed at 6 to 8 μM for up to 72 h (Fig. 1A). The IC50 were 7.0 and 6.5 μM in 24 h in PC3 and DU145 cells, respectively. No further inhibitory effects were observed with higher doses of solamargine. Similar result has been shown in an additional CRPC cell line C4-2B cells with IC50 3.277 μM in 24 h (Fig. 1A). Note that solamargine had little effects on cell growth at the doses showed significant effects on prostate cancer cells in one human benign prostate hyperplasia epithelial cells (BPH-1) (Fig. 1A). Moreover, the cell cycle phase distribution of CRPC cells treated with increased doses of solamargine for 24 h was analyzed by flow cytometry. We observed that, compared with the untreated control cells, solamargine significantly increased the proportion of cells at G0/G1 and G2/M phases (from 52.1 to 69% and from 9.96 to 13.9%), while the proportion of cells at S phases were reduced (from 32.9 to 15%, and from 28.8 to 22.3%) in PC3 and C4-2B cells, respectively (Fig. 1B,C). Together, these findings suggested that solamargine inhibited cell growth and induced cell cycle arrest in CRPC cells.

Metformin, the most widely used drug in treatment of type 2 diabetes, has been shown to inhibit growth of different cancer types in several studies through AMPK-dependent and -independent signaling pathways^28^,^29^,^30^,^31^,^32^. Herein, we also asked whether combination of known AMPK activator metformin and solamargine could have enhanced effect. Interestingly, while metformin alone had little effect, combination of solamargine and metformin significant reduced the proliferation of CRPC cells (Fig. 1D). This implied a potential new mechanism by which the combination of solamargine and metformin enhanced the growth inhibition of prostate cancer cells. As expected, while compound C stimulated cell growth, it also resisted in part the inhibitory effect of solamargine on cell growth (Fig. 1E).

### Solamargine and metformin increased phosphorylation of AMPKα

**Figure 2:**
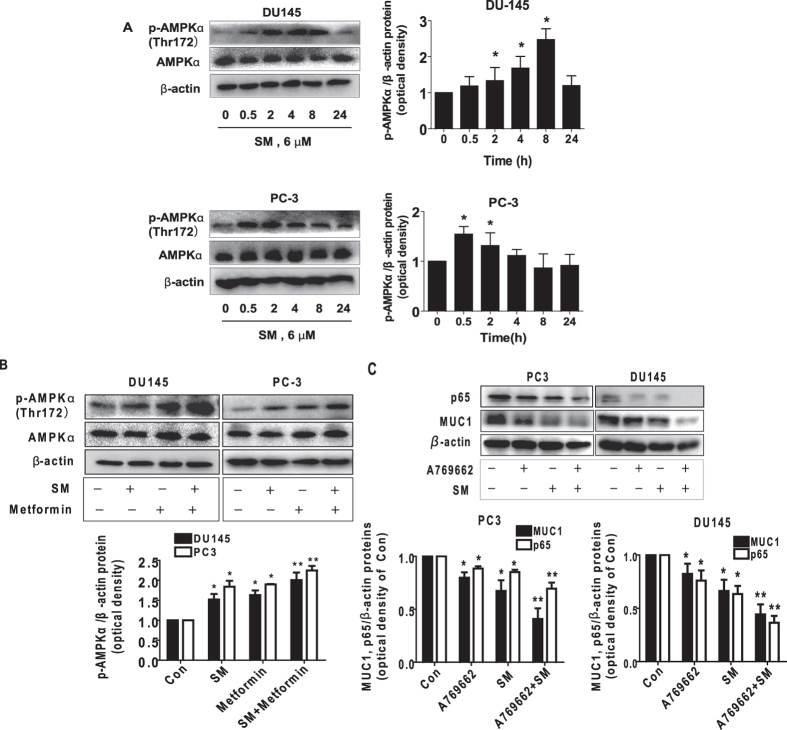
Solamargine and metformin increased phosphorylation of AMPKα. (**A**) PC3 and DU145 cells were treated with solamargine (6 μM) in the indicated times, and cell lysate was harvested and the expression of the phosphorylated and total protein of AMPKα was measured by Western blot analysis using corresponding antibodies. GAPDH was used as loading control. (**B**) PC3 and DU145 cells were treated with solamargine (6 μM) and metformin (5 mM) for 2 h. Afterwards, the phosphorylation and expression of AMPKα were detected by Western blot. The figures are representative cropped gels/blots that have been run under the same experimental conditions. (**C**) PC3 and DU145 cells were treated with solamargine (6 μM) and A-769662 (100 μM, Obtained from Selleck Chemicals, Houston, TX, USA) for 24 h. Afterwards, the protein expressions of p65 and MUC1 were detected by Western blot. Values in bar graphs were given as the mean ± SD from three independent experiments performed in triplicate. *Indicates significant difference as compared to the untreated control group (P < 0.05). **Indicates significant difference from the solamargine treated alone (P < 0.05).

Because of the controversial observations of AMPK signaling in pro- or anti-tumorigenic roles^22^,^33^ and the results from the effect of metformin in our study, we then assessed the effect of solamargine on activation of AMPK in this process. We showed that solamargine increased the phosphorylation of AMPKα in a time-dependent fashion with significant induction observed at 2–8 h and 0.5–2 h in PC3 and DU145 cells, respectively (Fig. 2A). Furthermore, combination of solamargine and metformin further increased the phosphorylation of AMPKα in PC3 and DU145 cells (Fig. 2B). Similar results were observed using A-769662, an AMPK direct activator^34^ in combining with solamargine (Fig. 2C). Together, this implied an AMPK-dependent signaling in this process.

### The effects of solamargine and metformin on protein expression of MUC1 through activation of AMPKα

**Figure 3:**
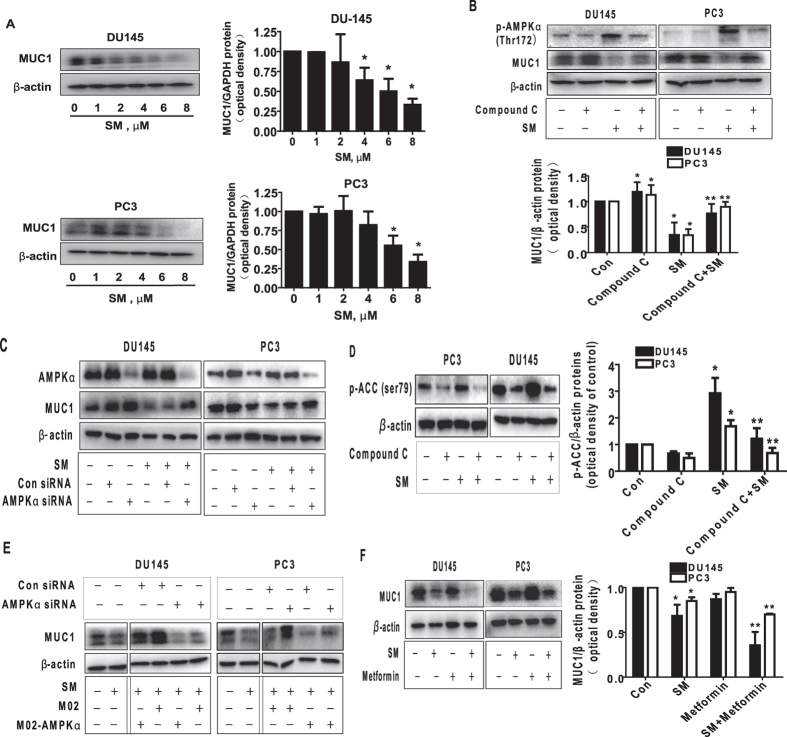
The effect of solamargine and metformin on protein expression of p65 and MUC1 through activation of AMPKα. (**A**) PC3 and DU145 cells ells were exposed to increased concentration of solamargine (6 μM) for 24 h. Afterwards, the expression of MUC1 proteins was detected by Western blot. (**B**) PC3 and DU145 cells were treated with compound C (10 μM) for 2 h before exposure of the cells to solamargine (6 μM) for an additional 2 h. Afterwards, the expression of MUC1 protein were detected by Western blot using antibodies against MUC1. (**C**) PC3 and DU145 cells were transfected with the control or AMPKα siRNA (50 nM) for 24 h before exposing the cells to solamargine for an additional 24 h. Afterwards, AMPKα and MUC1 protein expression were determined by Western blot. (**D**) PC3 and DU145 cells were treated with compound C (10 μM) for 2 h before exposure of the cells to solamargine (6 μM) for an additional 2 h. Afterwards, the phosphorylation of acetyl-CoA arboxylase (ACC) were detected by Western blot. (**E**) DU145 and PC3 cells silenced of AMPKα by siRNA previously were transfected with control and AMPKα overexpression vector for 24 h before exposing the cells to solamargine (6 μM) for an additional 24 h. Afterwards, MUC1 protein expressions were determined by Western blot. Silencing of AMPKα was detected by Western Blot previously. (**F**) PC3 and DU145 cells were treated with solamargine (6 μM) and metformin (5 mM) for 24 h. Afterwards, the expression of MUC1 protein were detected by Western blot. The figures are representative cropped gels/blots that have been run under the same experimental conditions. Values in bar graphs were given as the mean ± SD from three independent experiments performed in triplicate. *Indicates significant difference as compared to the untreated control group (P < 0.05). **Indicates significant difference from solamargine treated alone (P < 0.05).

Although AMPK-independent effects have been reported^31^,^32^, Metformin acts through its effect on AMPK-dependent signaling have also been shown in several studies^28^,^29^,^30^. Because of this, we next examined the relevant molecular mechanism by solamargine in the presence or absence of metformin. We showed that solamargine reduced the protein expression of MUC1, a membrane-anchored mucin, in the dose-dependent manner in PC3 and DU145 cells (Fig. 3A). Interestingly, the inhibitor of AMPK (compound C) not only inhibited but also reversed the effect of solamargine on MUC1 protein expression within the 24 h treatment (Fig. 3B). Similar results were observed in cells silencing of AMPKα gene by siRNA methods (Fig. 3C). As expected, we found that compound C blocked the solamargine-induced phosphorylation of acetyl-CoA carboxylase (ACC), one of the known downstream targets of AMPK^35^, confirming the efficacy of compound C in this process (Fig. 3D). Moreover, exogenously expressed AMPKα was found to restore the effect of solamargine on MUC1 protein expression in both cell lines silencing of endogenous AMPKα gene (Fig. 3E). Together, the results above confirmed a critical role of AMPKα in this process. Furthermore, we observed that combination of solamargine and metformin further reduced the protein expression of MUC1 suggesting an additive effect in this process (Fig. 3F).

### Solamargine reduced mRNA and promoter activity of MUC1

**Figure 4:**
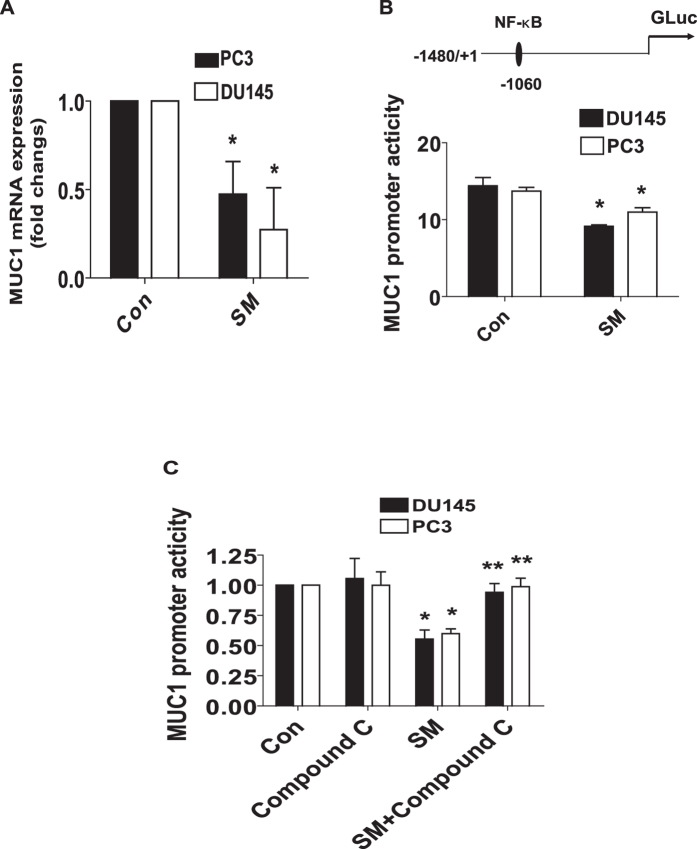
Solamargine inhibited mRNA and promoter activity of MUC1 through activation of AMPK. (**A**) PC3 and DU145 cells were exposed to solamargine (6 μM) for 24 h, followed by measuring the mRNA expressions of MUC1 by qRT-PCR. (**B**) PC3 and DU145 cells were transfected with wild type human MUC1 promoter reporter construct ligated to luciferase reporter gene and internal control secreted alkaline phosphatase for 24 h, followed by treating with solamargine for an additional 24 h. (**C**) PC3 and DU145 cells were treated with compound C (10 μM) for 2 h before transfecting the cells with wild type human MUC1 promoter reporter construct ligated to luciferase reporter gene and internal control secreted alkaline phosphatase for 24 h, followed by treating with solamargine (6 μM) for an additional 24 h. Afterwards, the promoter activities were determined using the Secrete-Pair Dual Luminescence Assay Kit as described in the Materials and Methods Section. *Indicates significant difference as compared to the untreated control group (P < 0.05). **Indicates significant difference from solamargine treated alone (P < 0.05).

Moreover, we showed that solamargine reduced mRNA levels of MUC1 as determined by quantitative real-time PCR (qRT-PCR) methods (Fig. 4A). In addition, we found that solamargine decreased promoter activity of MUC1 gene in PC3 and DU145 cells (Fig. 4B), and this was eliminated in the presence of compound C (Fig. 4C). The findings above indicated that the transcriptional reduction of MUC1 gene expression by solamargine was through the activation of AMPK signaling.

### Exogenous expression of p65 abrogated the effect of solamargine on MUC1 expression

**Figure 5:**
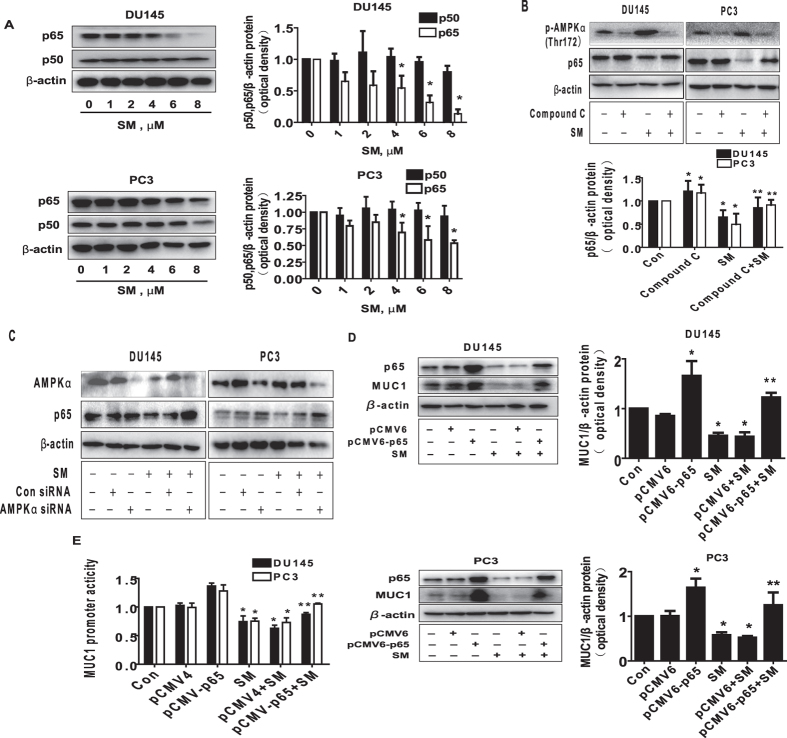
Exogenously expression of p65 abrogated the effect of solamargine on MUC1 expression. (**A**) PC3 and DU145 cells ells were exposed to increased concentration of solamargine (6 μM) for 24 h. Afterwards, the expression of p65 and p50 proteins was detected by Western blot. (**B**) PC3 and DU145 cells were treated with compound C (10 μM) for 2 h before exposure of the cells to solamargine for an additional 2 h. Afterwards, the expression of p65 protein were detected by Western blot. (**C**) PC3 and DU145 cells were transfected with the control or AMPKα siRNA (50 nM) for 24 h before exposing the cells to solamargine for an additional 24 h. Afterwards, AMPKα and p65 protein levels were determined by Western blot. (**D**) PC3 and DU145 cells were transfected with the control (pCMV4) or expression construct of p65 for 24 h before exposing the cells to solamargine for an additional 24 h. Afterwards, p65 and MUC1 protein expression were determined using Western blot. (**E**) PC3 and DU145 cells were transfected with the control (pCMV4) or expression construct of p65, and wild type human MUC1 promoter reporter construct ligated to luciferase reporter gene and internal control secreted alkaline phosphatase for 24 h before exposing the cells to solamargine (6 μM) for an additional 24 h. Afterwards, the Luciferase reporter activity was measured using Luciferase Assay System as described in the Materials and Methods Section. The figures are representative cropped gels/blots that have been run under the same experimental conditions. Values in bar graphs were given as the mean ± SD from three independent experiments performed in triplicate. *Indicates significant difference as compared to the untreated control group (P < 0.05). **Indicates significant difference from solamargine treated alone (P < 0.05).

Early study showed that MUC1 promoter region contained NF-κB/p65 binding site that mediated the MUC1 promoter activity and gene expression in normal and cancer cells^36^. This was for this reason we further explored the role of p65 in this process; we showed that solamargine reduced the protein expression of p65 in the dose-dependent manner in PC3 and DU145 cells (Fig. 5A). As expected, the inhibitor of AMPK (compound C) not only inhibited but also blocked the effect of solamargine on p65 protein expression within the 24 h treatment (Fig. 5B). Similar results were observed in cells silencing of AMPKα gene by siRNA methods (Fig. 5C). Note that solamargine had no effect on p50 protein expression (Fig. 5A). Moreover, exogenously expression of p65 overcame the effect of solamargine on MUC1 protein expression and promoter activity in PC3 and DU145 cells (Fig. 5D,E); this suggested that p65 is an upstream of MUC1, and that solamargine reduced expression of MUC1 through reduction of NF-κB/p65 signaling.

### While overexpression of MUC1 had no effect on p65, it attenuated the effect of solamargine on cell growth inhibition and phosphorylation of AMPKα

**Figure 6:**
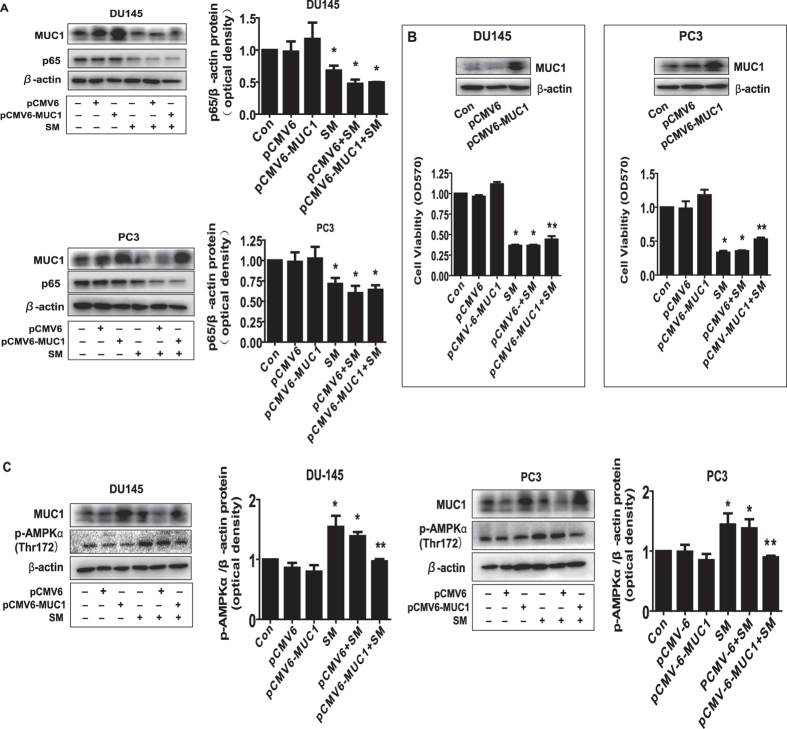
While overexpression of MUC1 had no effect on p65, it attenuated the effect of solamargine on cell growth inhibition and phosphorylation of AMPKα. (**A**) PC3 and DU145 cells were transfected with the control (pCMV6) or expression constructs of MUC1 for 24 h before exposing the cells to solamargine (6 μM) for an additional 24 h. Afterwards, p65 and MUC1 protein expression were determined by Western blot. (**B**) PC3 and DU145 cells were transfected with the control (pCMV6) or expression constructs of MUC1 for 24 h before exposing the cells to solamargine (6 μM) for an additional 48 h. Afterwards, the cell viability was determined using the MTT assay as described in the Materials and Methods Section. Insert on the upper panel represented the protein levels of MUC1 as determined using Western blot. GAPDH was used as internal control. (**C**) PC3 and DU145 cells were transfected with the control (pCMV6) or expression constructs of MUC1 for 24 h before exposing the cells to solamargine (6 μM) for an additional 8 h. Afterwards, p-AMPKα and MUC1 protein were determined by Western blot. The figures are representative cropped gels/blots that have been run under the same experimental conditions. Values in bar graphs were given as the mean ± SD from three independent experiments performed in triplicate. *Indicates significant difference as compared to the untreated control group (P < 0.05). **Indicates significant difference from solamargine treated alone (P < 0.05).

To further characterize the role of MUC1 in this process, we transfected the exogenously expressed MUC1 plasmid into the cells and found that, while overexpression of MUC1 had no effect on solamargine-reduced p65 protein expression in PC3 and DU145 cells (Fig. 6A); it significantly antagonized the effect of solamargine on cell growth inhibition (Fig. 6B). This result indicated a critical role of MUC1 in this process suggesting reduction of MUC1 expression at least in part mediated the solamargine-inhibited cell growth. Interestingly, exogenously expression of MUC1 attenuated the solamargine-induced phosphorylation of AMPKα in PC3 and DU145 cells (Fig. 6C).

### ***In vivo*** anti-tumor activity of solamargine

**Figure 7:**
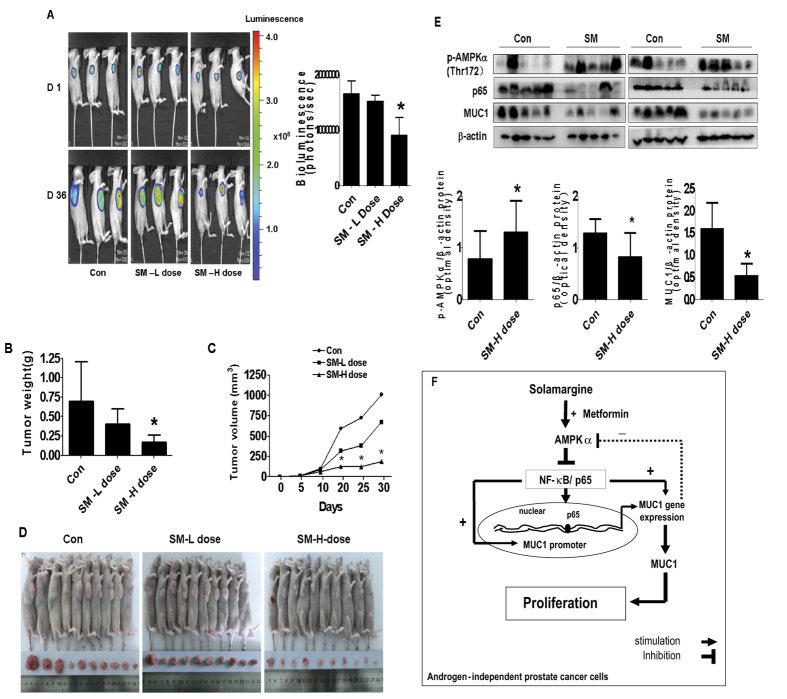
The effect of solamargine treatment in the xenograft mice model. Mice (n = 10/group) were divided to 3 groups [Con (saline), Low (L, 5 mg/kg) and High (H, 10 mg/kg) doses], and solamargine was given around the 10^th^ day after tumor cells injection by gavages daily for up to 30 days. (**A**) The xenografts were assessed by *in vivo* bioluminescence imaging at the end of the experiments (on day 30). The tumor growth was monitored by injecting luciferin in the mice followed by measuring bioluminescence using IVIS Imaging System. Imaging and quantification of signals were controlled by the acquisition and analysis software living image as described in the Materials and Methods section. Representative images are shown. (**B**,**C**) The xenografts were harvested on day 36, and the volume and weight of tumors were measured. The bar graphs represented the tumor weight and volume of mice results of as mean ± SD from three independent experiments. (**D**) The photographs of solamargine or vehicle-treated xenografts derived from nude mice are shown. (**E**) At the end of the experiments, xenograft tumors were isolated from individual animals and the corresponding lysates were processed for detecting p65, MUC1 and p-AMPKα by Western blot. GAPDH was used as loading control. Values in bar graphs were given as the mean ± SD from three independent experiments *Indicates the significant difference from untreated control (p < 0.05). (**F**) The diagram shows that solamargine inhibits the growth of androgen-independent prostate cancer cells through AMPKα-mediated inhibition of p65, followed by reducing expression of MUC1 gene. There is a synergy of solamargine and metformin. The feedback regulatory loop of AMPKα signaling pathway further demonstrates the critical role of MUC1 in contributing to the overall responses of solamargine.

We also tested the effect of solamargine on tumor growth and expression of MUC1 in xenografted nude mouse model. Luciferase-expressing DU145 cells were injected subcutaneously in nude mice. Mice bearing xenografted tumor was treated by gavages once every other day for different doses of solamargine (5 and 10 mg/kg, respectively) for up to 36 days. We found that, compared to the control group, the high dose solamargine-treated mice showed a significant delayed tumor growth, without any severe adverse events, as assessed by the Xenogen IVIS200 System (Fig. 7A). The differences in the levels of luciferase expression correlates with the tumor area. In addition, we noticed a significant reduction of the tumor weight and volume in the high doses of solamargine treatment group as compared to the control group (Fig. 7B–D). By Western blot, fresh tumors harvested from the aforementioned experiment showed that solamargine efficiently decreased phosphorylation of AMPKα, p65 and MUC1 protein expressions *in vivo* in the high dose solamargine treatment group as compared to that in the control one (Fig. 7E).

## Discussion

CRPC shows limited responses to most treatment options. This therapeutic dilemma resulted in less progress in prolongation of patient survival and enhancing quality of life. On the other hand, many patients die of recurrent and secondary disease (metastases). Therefore, searching for new adjuvant therapeutic options or agents to supplement current therapeutic modalities becomes strongly needed. Solamargine is a promising anticancer agent for various cancer types with mechanistic involvement of multiple pathways and molecular targets^8^,^9^,^10^,^11^. There were less information regarding the effect of this agent on growth of prostate cancer cells, therefore, the molecular mechanism of controlling the growth of prostate cancer cells by this agent remain unknown. In this study, we observed a significant inhibition of growth of prostate cancer cells not only by solamargine alone, but also, more importantly an additive response by solamargine in combing with metformin, an oral anti-diabetic medication in CRPC cells. These findings implied that pathways other than AR-mediated were involved in this process. The does used in this study were consistent with others and showed significant effects on controlling cancer cell survival without toxicities^11^,^37^,^38^,.

In this study, we demonstrated the role of AMPK signaling pathway in mediating the effect of solamargine in controlling the growth of CRPC cells. The activation of AMPK by solamargine has never been shown in the past. Activation of AMPK were reported to be involved in the anti-tumor responses in several cancer types including prostate, suggesting the tumor suppressor role of this kinase^16^,^17^,^18^,^39^,^40^ although conflicting observations have also been shown^19^,^20^. Nevertheless, our results suggested that activation of AMPK signaling was involved in effect of solamargine in the controlling the growth of CRPC cells. thus, The utilization of AMPK or perhaps the regulation of downstream targets may provide potential therapeutic targets in the treatment of prostate cancer^41^. Of note, in line with our results, the compound C directly inhibition of the phosphorylation of AMPK itself have been reported in several other studies^42^,^43^,^44^. Concerning an additional feedback role, the effects of compound C on AMPK signaling could be more complicated then we thought. Thus, more experiments are required to further confirm this in the future.

To further explore the potential mechanism underlying the inhibitory effect of solamargine, we tested the role of MUC1. Studies have highlighted the increased expression of MUC1 and its role in tumorigenesis in various cancer types including prostate cancer^27^,^45^,^46^. Our results illustrated both transcriptional and translational regulations of MUC1 by solamargine, and demonstrated a critical role of MUC1 expression in mediating the effect of solamargine on inhibition of growth of CRPC cell. This suggested that MUC1 could be a novel target of solamargine in the treatment of this type of malignancy. Furthermore, we observed an important role of NF-κB/p65 that may involve in the inhibitory effects of solamargine on MUC1 expression and growth of prostate cancer cells. The MUC1 C-terminal domain (MUC1-C) was associated with NF-κB/p65 in cancer cells, and the formation of MUC1-C and NF-κB/p65 complex showed to enhance nuclear translocation of NF-κB/p65, this in turn resulted in the consequently stimulated cancer cell growth and invasion^23^. On the other hand, MUC1-C was shown to bind to NF-κB/p65 and led recruitment of MUC1-C/NF-κB complexes to the promoters of NF-κB target genes, such as Bcl-xL and the promoter of the *MUC1* gene itself, in breast cancer and leukemia cells suggesting MUC1 was a direct activator of NF-κB/p65^47^. Our results suggested that MUC1 was downstream of NF-κB/p65 and that p65 influenced MUC1 expression both at transcriptional and translational levels. Consistent with this; one study showed that MUC1 promoter had NF-κB binding sites that involved in the cytokines-induced MUC1 expression in breast cancer cells^36^. Thus, we reasoned that the function and regulation of MUC1/NF-κB/p65 complexes are more complicated than we thought. Of note, our results implied that reduction of MUC1 expression was needed at least in part in mediating the inhibitory effect of solamargine on cell growth inhibition. Because of the fact that the overcoming effect of MUC1 expression on blockade of solamargine-inhibited cell growth was not so evident, we predicted that other downstream targets may also be involved in this process. Thus, more studies are required to further elucidate this.

We also observed the involvement of activation of AMPK signaling pathway in the regulation of p65 and MUC1. The links of AMPK to the regulation of NF-κB and MUC1 have been shown in other studies, suggesting that blockade of AMPK signaling affected NF-κB and reduced expression of MUC1 in several cell systems^48^,^49^,^50^. The AMPK has been reported to regulate the NF-κB signaling through distinct mechanisms^49^,^51^,^52^. However, the true role of AMPK signaling in regulation of NF-κB, such as whether targeting at transcriptional or/and translational levels, or via epigenetic regulatory pathways in CRPC cells, still required to be determined in the future. More interestingly, we also showed a negative feedback regulation of AMPK by MUC1. The feedback bidirectional circuit unveiled potential new regulatory mechanism by which solamargine inhibited growth of CRPC cells. This demonstrates relative common reciprocal physiopathological phenomenon of this kinase signaling^53^. Recent report suggested a reciprocal feedback mechanism involving AMPK activation and phospholipase D, a downstream target of the guanosine triphosphatase (GTPase) Ras homolog enriched in brain (Rheb), mediated by mammalian target of rapamycin (mTOR) signaling in cancer cells with therapeutic potential^54^. We believe that more studies are required to understand the in-depth mechanism for this feedback regulatory axis.

Furthermore, our results unveiled an additive effect of combination of solamargine and metformin in the inhibition of p65, MUC1 and prostate cancer cell growth, implying the potential new role and molecular mechanism in combination of solamargine and metformin in controlling CRPC cell growth. Metformin, a potential anticancer agent, has been shown to inhibit growth and induce apoptosis though AMPK-dependent and -independent signaling pathways in CRPC cells^55^,^56^. One study found that combination of metformin with other therapeutic agents, such as anti-AR agent bicalutamide, enhanced the growth inhibition of CRPC via AR-mediated signaling^57^. While metformin has been shown to inhibit NF-κB signaling in several other studies^58^,^59^, the regulation of metformin to MUC1 signaling remains elusive^60^. In the current study, metformin alone had no significant effect on MUC1 expression; we reasoned that metformin sensitized the therapeutic effect of solamargine in prostate cancer cell growth partly through enhancing the inhibition of MUC1 expression. Whether this was acted through AMPK-dependent pathway required to be determined. The combined findings of solamargine and metformin suggested an additive effect existed in our system, which may not be considered as a synergy because of the moderate effects. Thus, the true statistical significance specifically for the potential synergy needs to be determined.

More importantly, our *in vivo* data were consistent with the findings from that *in vitro*, confirming the effect of solamargine on prostate cancer growth inhibition and regulation of p65 and MUC1 expression^61^,^62^. The doses of solamargine used were based on our a series of experiments *in vivo* and other study^63^. In fact, there was scarce information available for the use of solamargine *in vivo* study. We believed that additional experiments are needed to further confirm this. On the other hand, through calculation, even through the doses used *in vivo* had high micromolar concentrations than that used *in vitro*, given the fact that this was via subcutaneous injection, thus the actual doses in animal blood expected to be much low although no reported information were available at this time. We think that further experiments are required to obtain this. Moreover, more studies are also needed to further determine the critical role of MUC1 in this process using cells stable transfected with shRNAs or/and exogenously expression vectors containing the coding region of full length MUC1 gene in nude mice model. In addition, whether solamargine has potential in prolonging the survival and inhibiting metastasis in prostate xenograft tumors, and more importantly, the synergy of solamargine and metformin in influencing the gene expression and tumor growth required to be elucidated.

Overall, our results show that solamargine inhibits the growth of CRPC cells through AMPKα-mediated reduction of NF-κB subunits p65, followed by reducing expression of MUC1 gene both *in vitro* and *in vivo*. More importantly, there are additive effects of solamargine and metformin with greater potency. The negative feedback regulatory loop of AMPKα further demonstrates the critical role of MUC1 in contributing to the overall responses of solamargine (Fig. 7F). This study unveils the novel mechanism by which solamargine alone or combination of solamargine and metformin inhibits or enhances the effect of inhibition of growth of CRPC cells.

## Materials and Methods

### Cell culture and chemicals

The CRPC cell lines DU145, PC3 and C4-2B, and one benign prostate hyperplasia epithelial cell line (BPH-1) were obtained from the (Sun Yat-sen Memorial Hospital, Sun Yat-sen University, Guangzhou, Guangdong Province, China). Cells were grown in F12K or DMEM (1:1) medium (obtained from GIBCO, Life Technologies, Grand Island, NY, USA) with supplemented 10% fetal bovine serum. Lipofectamine 3000 reagent was purchased from Invitrogen (Shanghai, China). The polyclonal antibody against MUC1 was obtained from Abcam (Cambridge, MA, USA). The antibodies against p65, p50, total AMPK and the phosphor-form (Thr-172), and phosphor-ACC (ser79) were purchased from Cell Signaling Technology Inc (Beverly, MA, USA). A769662, an known activator of AMPK, was obtained from Selleck Chemicals, Shanghai, China). Metformin, compound C (AMPK inhibitor) were obtained from Sigma-Aldrich (St. Louis, MO, USA) unless otherwise indicated.

### Western blot analysis

The detailed procedure was reported previously^64^. Briefly, protein concentrations were determined by the Bio-Rad protein assay. Equal amounts of protein from whole cell lysates were solubilized in SDS-sample buffers and separated on SDS polyacrylamide gels. Membranes were incubated with antibodies against MUC1, p65, p50, the phosphor and total AMPKα. The membranes were washed and incubated with a secondary goat antibody raised against rabbit IgG conjugated to horseradish peroxidase (Cell Signaling Technology, Inc., Beverly, MA, USA). The membranes were washed again and transferred to freshly made ECL solution (Immobilon Western; Millpore, Billerica, MA, USA), followed by observing the signals under the Molecular Imager ChemiDoc XRS Gel Imagine System (Bio-Rad, Hercules, CA, USA).

### Quantitative real-time PCR

A qRT-PCR assay was developed for the detection and quantification of MUC1 transcript. The primers used in this study were designed as follows: MUC1 forward 5′-ACGTCAGCGTGAGTGATGTG-3′; reverse 5′-GACAGACAGCCAAGGCAATG-3′; GAPDH forward 5′-AAGCCTGCCGGTGACTAAC-3′; reverse 5′-GCGCCCAATACGACCAAATC-3′, which used as an endogenous control. First-strand cDNA was synthesized by reverse transcription using oligo-dT primers and Superscript II reverse transcriptase according to the manufacturer’s protocol (Invitrogen, Grand Island, NY, USA). qRT-PCR was performed in a 20 μL mixture containing 2 μL of the cDNA preparation, 10 μL 2X SYBR Green Premix ExTaq (Takara), and 10 μM primer on an ABI 7500 Real-Time PCR System (Applied Biosystems, Grand Island, NY, USA). The PCR conditions were as follows: 10 min at 92 °C, followed by 30 cycles of 15 s at 92 °C, and 1 min at 65 °C. Each sample was tested in triplicate. Threshold values were determined for each sample/primer pair, the average and standard errors were calculated. The raw data were normalized to GAPDH and presented as relative MUC1 gene expression.

### Cell viability assay

Cell viability was measured using the 3-(4,5-dimethylthiazol-2-yl)-2, 5-diphenyltetrazolium bromide (MTT) assay as described previously^64^. In brief, prostate cancer cells were counted and seeded in a 96-well microtitre plate. The cells were treated with increasing concentrations of solamargine for up to 72 h. Afterwards, 20 μL MTT solution (5 g/L) was added and prostate cancer cells were incubated at 37 °C for additional 4 h. Supernatant was removed, and 200 μL solvent dimethyl sulfoxide was added to each well, and oscillated for 5–10 min. ELISA reader (Perkin Elmer, Victor X5, USA) was used to determine the optical density at 490 nm of absorbance. Cell viability was calculated as (absorbance of test sample/absorbance of control).

### Cell cycle analysis

The detailed procedure was reported previously^64^. Prostate cancer cells were cultured in 6-well plastic plates at 3 × 10^5^ cells/well and treated with increased doses of solamargine for 24 h. Afterwards, the cells were washed and resuspended in 500 μL mixture of PBS and ethanol (1.5 mL) for 2 h at 4 °C. Afterwards, the fixed cells were incubated in 1 mL of 0.1% sodium citrate containing propidium iodide (PI) and RNase for 30 min. The flow cytometry (FC500, Beckman Coulter, FL, USA) analysis were performed to detect the cell cycle, and the proportion of cells within the G0/G1, S, and G2/M phases of the cell cycle were analyzed using the MultiCycle AV DNA Analysis software (Phoenix Flow Systems, Inc. San Diego, CA,USA).

### Treatment with AMPKα siRNA

The detailed procedure was reported previously^65^. In brief, cells were seeded in 6-well or 96-well culture plates in RPMI 1640 medium containing 10% FBS (no antibodies), grown to around 60–80% confluence, and transiently transfected with AMPKα and control siRNAs (up to 50 nM) purchased from Santa Cruz Biotechnology, Inc (Dallas, Texas, USA) using Lipofectamine 3000 according to the manufacturer’s instructions, and incubated for up to 24 h. Afterwards, the cells were resuspended in the presence of solamargine for the indicated time for all other subsequent experiments.

### Transient transfection assay

This procedure was reported previously^66^. The control, MUC1 overexpression vectors were obtained from OriGene Technologies, Inc. (Rockville, MD, USA). The control and AMPKα expression vector (M02-AMPKα) were obtained from the GeneCopoeia, Inc. (Rockville, MD, USA). The control and p65 overexpression vector (pCMV4-p65) was obtained from the Addgene (Plasmid #21966)^67^. Briefly, cells were seeded in 6-well dishes and grown to 50–60% confluence. For each well, 2 μg of control, MUC1, AMPKα and p65 plasmid DNA constructs were transfected into the cells using Lipofectamine 3000 Transfection Reagent (Invitrogen, Shanghai, China) for up to 24 h, followed by treating with solamargine for an additional 24 or 48 h. In the separated experiments, control and wild type pEZX-PG04-MUC1 promoter constructs (purchased from GeneCopoeia, Inc., Rockville, MD, USA) with or without 0.2 μg of the internal control secreted alkaline phosphatase (SEAP) were co-transfected into the cells with the Lipofectamine 3000 Transfection Reagent. The preparation of cell extracts and measurement of luciferase activities were determined using the Secrete-Pair™ Dual Luminescence Assay Kit (GeneCopoeia, Inc).

### Tumor xenograft studies

Animal experiments were performed in accordance with guidelines for Care and Use of Laboratory Animals and the protocols were approved by Institutional Animal Care and Use Committee Animal Care of Guangdong Provincial Hospital of Chinese Medicine. A total of 30 female nude mice (eight-week-old) were obtained from Guangdong Provincial Research Center for Laboratory Animal Medicine (Foshan, Guangdong, China) and maintained at the Animal Center of Guangdong Provincial Hospital of Chinese Medicine in a specific pathogen-free environment with food and water provided. DU145 cells carrying luciferase report gene (DU145-Luc, obtained from the Guangzhou Land Biological Technology Co., Guangzhou, China) (5 × 10^6^ cells) were injected subcutaneously into nude mice. Mice were randomly assigned to three experimental groups [Con, low dose (5 mg/kg) and high doses (10 mg/kg) of solamargine] and xenografts were allowed to grow for over one week when tumors were detectable with calipers before treatment by gavages.

For bioluminescence imaging (BLI) procedure, xenografted tumors were monitored by noninvasive optical imaging on an IVIS200 Imaging System (Xenogen, Alameda, CA, USA). Animals were immobilized by inhalation of 2% isoflurane/O_2_ at the beginning and the end of treatment, followed by injecting peritoneal with 150 mg/kg D-luciferin in 200 μL (Xenogen; PerkinElmer, Waltham, MA, USA) and imaged 15 minutes later. The intensity of BLS in the luminescent area of the tumor was determined using the IVIS-200 Imaging System and reported as photons/sec. Tumor volume measurements were calculated using the formula for an oblong sphere: volume = (width^2^ × length). The body weights of the mice were measured once a week. All mice were euthanized on 36 days. The corresponding xenograft tumors were removed and processed for detecting the phosphorylation of AMPKα, p65 and MUC1 protein by Western blot.

### Statistical analysis

All experiments were repeated a minimum of three times. All data were expressed as means ± SD. Differences between groups were assessed by one-way ANOVA and significance of difference between particular treatment groups was analyzed using Dunnett’s multiple comparison tests (GraphPadPrism5.0 software, LaJolla, CA, USA). The results in graphs were presented relative to the control. Asterisks shown in the figures indicate significant differences of experimental groups in comparison with the corresponding control one (P < 0.05, see figure legends).

## Additional Information

**How to cite this article**: Xiang, S.T. *et al*. Activation of AMPKα mediates additive effects of solamargine and metformin on suppressing MUC1 expression in castration-resistant prostate cancer cells. *Sci. Rep*. **6**, 36721; doi: 10.1038/srep36721 (2016).

**Publisher’s note:** Springer Nature remains neutral with regard to jurisdictional claims in published maps and institutional affiliations.
